# Increasing Negativity of Age Stereotypes across 200 Years: Evidence from a Database of 400 Million Words

**DOI:** 10.1371/journal.pone.0117086

**Published:** 2015-02-12

**Authors:** Reuben Ng, Heather G. Allore, Mark Trentalange, Joan K. Monin, Becca R. Levy

**Affiliations:** 1 Yale School of Public Health, New Haven, Connecticut, United States of America; 2 Yale School of Medicine, New Haven, Connecticut, United States of America; Cardiff University, UNITED KINGDOM

## Abstract

Scholars argue about whether age stereotypes (beliefs about old people) are becoming more negative or positive over time. No previous study has systematically tested the trend of age stereotypes over more than 20 years, due to lack of suitable data. Our aim was to fill this gap by investigating whether age stereotypes have changed over the last two centuries and, if so, what may be associated with this change. We hypothesized that age stereotypes have increased in negativity due, in part, to the increasing medicalization of aging. This study applied computational linguistics to the recently compiled Corpus of Historical American English (COHA), a database of 400 million words that includes a range of printed sources from 1810 to 2009. After generating a comprehensive list of synonyms for the term *elderly* for these years from two historical thesauri, we identified 100 collocates (words that co-occurred most frequently with these synonyms) for each of the 20 decades. Inclusion criteria for the collocates were: (1) appeared within four words of the elderly synonym, (2) referred to an old person, and (3) had a stronger association with the elderly synonym than other words appearing in the database for that decade. This yielded 13,100 collocates that were rated for negativity and medicalization. We found that age stereotypes have become more negative in a linear way over 200 years. In 1880, age stereotypes switched from being positive to being negative. In addition, support was found for two potential explanations. Medicalization of aging and the growing proportion of the population over the age of 65 were both significantly associated with the increase in negative age stereotypes. The upward trajectory of age-stereotype negativity makes a case for remedial action on a societal level.

## Introduction

A controversy exists about whether age stereotypes (beliefs about old people) are becoming more negative or positive over time. Some researchers contend that these stereotypes have become more positive as the older population becomes increasingly healthy, with improved medical interventions, and increasingly powerful, with the growth of aging-advocacy groups [1, 2]. Others argue that age stereotypes have become more negative over time [3]. For example, a historical analysis found evidence that during the late 1800’s, attitudes toward old age began a transformation “from ambivalence to disrespect and even to hostility” ([4], p. 54).

Part of the reason for this controversy about the direction of the age-stereotype trend is that no study has systematically examined it over more than 20 years. The goal of our study was to fill this gap by investigating whether age stereotypes have changed over the last two centuries and, if so, what may be associated with this change.

It is important to understand how a culture conceptualizes older persons, because age stereotypes have predicted how older adults are treated by, for example, work supervisors and health-care providers [5]. Furthermore, in line with Stereotype Embodiment Theory, the age stereotypes that older individuals assimilate from their culture have been found to predict their cognitive and physical health [6].

In the current study, we applied computational linguistics in a novel way to the Corpus of Historical American English (COHA), a recently compiled historical database of approximately 400 million words [7], which allowed us to study trends in age stereotypes across two centuries. The database, that includes all the words of approximately 100,000 texts published from 1810 to 2009, is 100 times larger than any other structured corpus of historical English, and draws equally from popular magazines, newspapers, fiction and non-fiction books across time [7]. According to Cultivation Theory, different forms of media provide valuable resources to study because they reflect the culture, as well as present images that can impact how society and its members think about themselves [8].

We hypothesized that our linguistic analysis would reveal that age stereotypes have become more negative over the last 200 years. Further, we predicted two factors would contribute to greater negativity of age stereotypes over time. The first is an increase in the tendency to medicalize the elderly; that is, to associate the elderly with physical health or illness. This arose from: the replacement of acute illness with chronic illness as the primary source of morbidity and mortality; the objectification of older adults as patients rather than as individuals with interesting life experiences; and the development of a profitable disability industry that has targeted older individuals [9–11].

The second factor that we hypothesized would contribute to greater negative age stereotypes over time is the growing proportion of individuals over the age of 65, as this could lead the younger generation to perceive the older generation as a burden that might drain limited resources [12] (even if this perception is not based on reality). This prediction is supported by a study of 26 countries by Löckenhoff and colleagues that found participants from countries with a higher proportion of adults above 65 years tended to espouse more negative age stereotypes [12]. Our study built on this research by focusing on one country over time and by using a novel implicit measure of age stereotypes to examine whether the growing proportion of the population over age 65 contributes to the negativity of age stereotypes.

## Methods

This study used the Corpus of Historical American English (COHA), a database of approximately 400 million words that covers 1810–2009 (http://corpus.byu.edu/coha/). To examine age stereotypes in this database, we generated a comprehensive list of synonyms for the term *elderly* from 1810–2009 using two historical thesauri that included American English [13,14]. This generated a list of 11 *elderly* synonyms that appeared at least 10 times in the dataset. Some synonyms entered the database in later decades reflecting expected variation over time in language use (e.g., *senior citizen* first appeared in 1949). Three nouns (i.e., *aged*, *elderly*, *old people*) appeared across all 20 decades; these were analyzed as a subset.

After the *elderly* synonym list was generated, we identified the 100 words that co-occurred most frequently (referred to henceforth as collocates) with each of these *elderly* synonyms for each of the 20 decades. The inclusion criteria for the collocates were: (1) appeared within four words before or after the *elderly* synonym to represent lexical proximity [15]; (2) referred specifically to an old person (context checked by two raters); and (3) had a stronger association with the *elderly* synonym than other words that appeared in the database for that decade, as measured by a Mutual Information Score (MI) of three or greater, denoting semantic bonding between the *elderly* synonym and collocate [15–17]. A sample of 13,100 collocates met the study criteria. This is a novel application of concordance analysis, which is used in computational linguistics to examine language evolution [15–17], to the identification of stereotypes.

To generate the age-stereotype score per decade, each collocate that met the study criteria was rated on a scale from 1 *(very positive)* to 5 *(very negative)*, a method found to be reliable and valid to measure age-stereotype associated words [1,18]. For the two raters, the inter-rater reliability of the 11 words using Cronbach’s alpha was. 994 (95% CI:. 992, .996) for the scoring method. The medicalization of older persons was measured by rating each collocate on whether or not it related to physical health or illness. Examples of these collocates include *stamina* and *sickness*. The age-stereotype scores and medicalization-of-older-individuals scores for all collocates were summed for each *elderly* synonym and then weighted by the number of times it appeared in that decade. The percentage of the population above 65 years was calculated for each of the relevant decades based on United States Census Bureau reports [19, 20].

## Results

**Fig 1:**
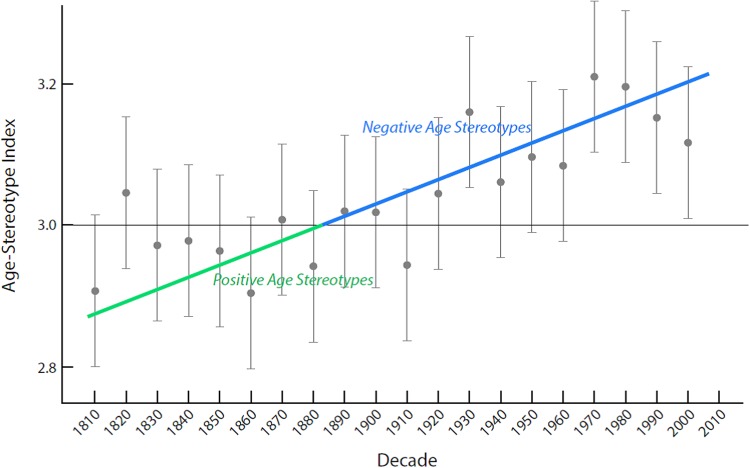
Increasing negativity in age stereotypes of older adults from 1810–2009 with best-fit line and 95% confidence limits for each decade. The horizontal line indicates the neutral point in the Age-Stereotype Index, with scores lower than three (before 1880) indicating average positive-age-stereotype scores and scores greater than three (after 1880) indicating average negative-age-stereotype scores.

As expected, we found that age stereotypes became significantly more negative over time, β = .01, p<.0001 in the full 11-*elderly*-synonym sample (Fig. 1). The intercept was 2.93 (p<.0001; 95% CI: 2.88, 2.98), indicating that age stereotypes started slightly positive in 1810. The passing of each decade from 1810–2009 was associated with a. 012-unit (95% CI:. 008, .02) linear increase in negative age stereotypes; resulting in an 8.2% increase in negative age stereotypes over 20 decades.

Support was found for a linear trend of the increasingly negative age stereotypes in the 11-*elderly*-synonym sample. The significant linear model was the best fit for the age-stereotype scores. All age-stereotype ratings over 20 decades were within the 95% confidence interval of the trend line. In contrast to the linear model, neither the quadratic (β = -.003, p = .26) or cubic (β = .00008, p = .29) models of the age-stereotype scores were significant.

Similar to the 11-*elderly*-synonym sample, as expected, in the three-*elderly*-synonym sample, we found that age stereotypes became significantly more negative over time, β = .012, p = .0007. In this sample, the intercept was also 2.93 (p<.0001; 95% CI: 2.88, 2.98), indicating that age stereotypes started slightly positive in 1810. In this sample, each decade was associated with a. 012-unit (95% CI:. 006, .18) increase in negative age stereotypes; resulting in an 8.2% increase in negative age stereotypes over 20 decades.

Again, in the three-*elderly*-synonym sample, support was found for a linear trend of the increasingly negative age stereotypes. The significant linear model was the best fit for the age-stereotype scores; all ratings were within the 95% confidence interval of the linear trend line. In contrast to the linear trend, neither the quadratic (β = -.002, p = .47) or the cubic (β = .00008, p = .46) models were significant.

As can be observed in the Age-Stereotype-Index-average scores of Fig. 1, before 1880, the majority of decades, 75%, were rated as expressing positive age stereotypes, whereas after 1880, the majority of decades, 92%, were rated as expressing negative age stereotypes. Further, starting in the 1920’s, all the decades were rated as expressing negative age stereotypes.

Medicalization of aging (β = .03, p <.0001) was a significant predictor of the negativity of age stereotypes. Every 1% increase in medicalization collocates was associated with a. 03-unit (95% CI:. 02, .04) increase in negative age stereotypes per decade, or a 20.5% increase over 200 years in the 11-*elderly*-synonym sample. In the three-*elderly*-synonym sample, physical-health descriptors were also significant, (β = .04, p <.0001), with a slightly larger effect size: every 1% increase in medicalization collocates was associated with an increase in negativity of. 04 units (95% CI:. 03, .05) per decade, and 27.0% increase over 20 decades.

The second predicted contributing factor of the proportion of the population above 65 years was also significantly associated with negativity of age stereotypes, β = .019, p <.0001 in the full 11-*elderly*-person synonym sample across the 200 years. Results were similar in the three-*elderly*-person synonym sample, β = .02, p <.0001. In both datasets, every 1% increase in proportion of population above 65 years was associated with a. 02-unit (95% CI:. 01, .03) increase in negativity of age stereotypes per decade, and a 12.4% increase overall.

## Discussion

This study’s findings illustrate that the negativity of age stereotypes is on an upward trajectory in the United States. According to the trend line, age stereotypes were positive from 1810 to 1879, became neutral in 1880, and have become increasingly negative in the following decades.

Finding similar results in the trend toward more negative age stereotypes over 200 years in the two ways of sampling *elderly* synonyms increased our confidence in the pattern of findings. The sub-sample of three *elderly* synonyms had the advantage of including the same three words across all 20 decades. The full sample of 11 *elderly*-synonyms, which was a comprehensive set of all words that appeared in the historical thesaurus that met the study criteria, had the advantage of reflecting the range of ways an old person was referred to over the 200 years [21].

Future research should continue to examine the predictors of the linear trend toward greater negativity of age stereotypes in more detail, as the current study examined associations rather than causal relationships. It is likely that other factors also contribute to the increase in negativity, such as modernization with its associated industrialization, urbanization, and mobilization that reduces the number of children growing up near older role models [4, 11, 22, 23].

In conclusion, this study has methodological and cultural implications. Methodologically, the implicit technique used to study age stereotypes over decades by applying lexical proximity and semantic bonding, after checking context, could be applied to understanding wide-spread beliefs about a diversity of other issues. Culturally, as the proportion of old persons continues to rise, the negativity of age stereotype might continue on its upward trajectory. The linear nature of this trend suggests it is systemic. Therefore, only a societal-based campaign is likely to successfully combat the ageism that is expressed through the stereotypes. This could provide a likely benefit for all ages of the population.
